# Functional electrical stimulation therapy for restoration of motor function after spinal cord injury and stroke: a review

**DOI:** 10.1186/s12938-020-00773-4

**Published:** 2020-05-24

**Authors:** Cesar Marquez-Chin, Milos R. Popovic

**Affiliations:** 1 Kite Research Institute, Toronto Rehabilitation Institute-University Health Network, 550 University Avenue, Toronto, ON M5G 2A2 Canada; 2 Institute of Biomaterials and Biomedical Engineering, University of Toronto, Toronto, ON Canada; 3 Center for Advancing Neurotechnological Innovation to Application, CRANIA, University Health Network, Toronto, ON Canada

**Keywords:** Functional electrical stimulation, Neuroprosthesis, Neurorehabilitation, Stroke, Spinal cord injury

## Abstract

Functional electrical stimulation is a technique to produce functional movements after paralysis. Electrical discharges are applied to a person’s muscles making them contract in a sequence that allows performing tasks such as grasping a key, holding a toothbrush, standing, and walking. The technology was developed in the sixties, during which initial clinical use started, emphasizing its potential as an assistive device. Since then, functional electrical stimulation has evolved into an important therapeutic intervention that clinicians can use to help individuals who have had a stroke or a spinal cord injury regain their ability to stand, walk, reach, and grasp. With an expected growth in the aging population, it is likely that this technology will undergo important changes to increase its efficacy as well as its widespread adoption. We present here a series of functional electrical stimulation systems to illustrate the fundamentals of the technology and its applications. Most of the concepts continue to be in use today by modern day devices. A brief description of the potential future of the technology is presented, including its integration with brain–computer interfaces and wearable (garment) technology.

## Background

Losing the ability to move voluntarily can have devastating consequences for the independence and quality of life of a person. Stroke and spinal cord injury (SCI) are two important causes of paralysis which affect thousands of individuals around the world. Extraordinary efforts have been made in an attempt to mitigate the effects of paralysis. In recent years, rehabilitation of voluntary movement has been enriched by the constant integration of new neurophysiological knowledge about the mechanisms behind motor function recovery. One central concept that has improved neurorehabilitation significantly is *neuroplasticity*, the ability of the central nervous system to reorganize itself during the acquisition, retention, and consolidation of motor skills [1]. In this document, we present one of the interventions that has flourished as a consequence of our increased understanding of the plasticity of the nervous system: functional electrical stimulation therapy or FEST. The document, which is not a systematic review, is intended to describe early work that played an important historical role in the development of this field, while providing a general understanding of the technology and applications that continue to be used today. Readers interested in systematic reviews of functional electrical simulation (FES) are directed to other sources (e.g., [2–4]).

### Stroke

Stroke is the fifth cause of death in the United States and a leading cause of disability [5]. It is a localized death of brain tissue following an interruption of blood supply. A stroke caused by a ruptured blood vessel is often referred to as a *hemorrhagic stroke*, while one produced by a blockage of a blood vessel is an *ischemic stroke*. The majority (87%) of strokes are ischemic [5]. The location and extent of the necrosis determine the effects of the stroke, which can affect behavior, emotion, communication, and voluntary movement, among other things. A common effect of stroke is *hemiplegia* in which the ability to move one side of the body is impaired. This condition can range from mild, in which the decrease in motor function is barely noticeable, to severe, in which the ability to move is greatly impaired or completely lost. Recovery has been historically considered to peak at six months after stroke with decreasing probability of observing improvements afterward.

### Spinal cord injury

Spinal cord injury (SCI) occurs when the spinal cord is damaged leading to a loss of sensory and/or motor function. The spinal cord is part of the central nervous system and it is composed of a major bundle of nerves that allow communication between the brain and the rest of the body (i.e., through peripheral nerves). It also contains neuronal structures (grey matter) responsible for monosynaptic and polysynaptic reflexes, as well as for carrying out tasks such as bladder and bowel voiding, and locomotion. SCI often affects the body bilaterally and can be *traumatic* (e.g., resulting from a motor vehicle accident) or *non*-*traumatic* (e.g., due to a tumor). SCI can be complete or incomplete according to the extent of the damage to the spinal cord. The level of SCI is important as sensory and/or motor impairment takes place below the level of injury, with higher lesions affecting a greater proportion of the body. In the context of voluntary motor function, a lesion of the lumbar and thoracic levels can result in *paraplegia*, which affects trunk and lower extremity function. An SCI at the cervical level can, in addition, affect the capacity to move the upper limbs, a condition known as *tetraplegia*.

### Rehabilitation after stroke and SCI

Recovering voluntary motor function can improve the independence and quality of life after stroke and SCI. Therapy can focus on multiple aspects including, for example, increasing strength and range of motion. Recent interventions that integrate new knowledge on the neurological mechanisms behind recovery of movement have emerged. Of particular importance has been the concept of neuroplasticity, the nervous system’s ability to modify its synaptic connectivity to reorganize itself and incorporate new motor abilities. This document describes functional electrical stimulation therapy (FEST), an intervention that takes advantage of neuroplasticity to restore the ability to perform voluntary movement after stroke and SCI.

## Functional electrical stimulation

Electrical current can elicit a response in excitable cells including neurons. Devices that can deliver controlled discharges have made it possible to assist individuals with different medical conditions. Cochlear implants to restore hearing, phrenic pacemakers that assist respiration, systems to void the bladder, cardiac pacemakers to ensure cardiac function, and deep brain stimulation to control tremor due to Parkinson’s disease are examples of applications of electrical stimulation systems.

### Neuromuscular stimulation

*Neuromuscular stimulation* (*NMES*) is one application of electrical stimulation used in rehabilitation of movement. In it, electrical stimulation produces contractions of paralyzed muscles that are still innervated [6]. NMES can increase the patients’ participation in voluntary activities by reducing impairment. For example, NMES can be used to increase muscle strength, improve shoulder subluxation (dislocation), reduce muscle tone, and produce movement.

### Functional electrical stimulation

Functional electrical stimulation (FES) is a subtype of NMES in which the stimulation assists functional and purposeful movements. This is achieved by applying electrical stimulation to muscles that, when they contract, produce a movement that can be used functionally. Examples of functional movements include lifting a book from a desk, bringing a bottle of water to the mouth, and holding a pen to write. The muscles, as well as the sequence in which they contract, are selected specifically to produce the desired movement. An FES system that facilitates a specific movement is often referred to as a *neuroprosthesis* or motor neuroprosthesis. For example, a neuroprosthesis for grasping is an FES system that restores the ability to grasp objects. Other examples include neuroprostheses for standing, walking, reaching, as well as reaching and grasping, all of which will be described below.

### Components of a neuroprosthesis

The basic components of a neuroprosthesis are an electrical stimulator, electrodes that deliver the stimulation, sensors for user or automatic control of the stimulation, and in some cases, an orthosis that provides additional assistance to perform the desired movement [7].

#### Electrical stimulator

The electrical stimulator is responsible for generating the electrical discharges that produce muscle contractions. Delivery of the stimulation is achieved through individual stimulation channels. A stimulation channel consists of a pair of electrodes (cathode and anode) used to deliver complex stimulation pulses (important characteristics of the stimulation pulses are described below). A stimulator can have multiple stimulation channels, each of which can stimulate individual muscles using unique settings. A multichannel *programmable* stimulator, which allows specifying the sequence in which each channel is active, makes it possible to facilitate different functional movements [8].

#### Stimulation electrodes

Electrical stimulation can be delivered using electrodes with different levels of invasiveness; they can be completely or partially implanted, known as implanted and percutaneous electrodes, respectively, or can also be placed on the surface of the body (transcutaneous or noninvasive electrodes). Each type of electrode offers advantges and disadvantages with respect to their flexibility, stimulation specificity, usability, and cost. Table 1 displays a summary of the types of electrodes commonly used for stimulation.

**Table 1:** Stimulation electrodes with different levels of invasiveness

Type	Typical current	Advantages	Disadvantages
Invasive			
Implanted	25 mA	High stimulation specificity	Require surgery
		Suitable for long-term use	Placement cannot be modified after implantation
Percutaneous	25 mA	High stimulation specificity	Require surgery
		Suitable for short-term use	
Non-invasive			
Transcutaneous (surface)	2 mA–120 mA	Do not require surgery	Unsuitable for stimulation of deep muscles
		Easy to reposition	Often require higher stimulation current

##### Implanted electrodes

As the name suggests, implanted electrodes are inserted surgically into the body. They can be placed in close proximity to targeted nerves resulting in high selectivity (i.e., it is easier to isolate specific muscles to stimulate). They are better suited for long-term use. One risk is that the surgery required to implant electrodes can increase the risk of infection [8]. Another consideration is the cost involved in delivering FES using implanted electrodes [9]. The electrical current necessary to produce a muscle contraction with implanted electrodes is in the range of 25 mA [8]. Theoretically speaking, once implanted, FES systems that use implanted electrodes require less time to don and doff compared to surface stimulation technology; despite full implantation of the electrodes there are often external components that the user must don as part of the complete neuroprosthesis (e.g., a controller interface). However, recent advances with Bioness, MyndMove, and textile computing solutions (described below) challenge this long held premise; these new surface stimulation systems are at least as fast to don and doff as implanted systems. An important drawback of implanted electrodes is that, once implanted, their position cannot be readily changed.

##### Percutaneous electrodes

Percutaneous electrodes typically have the form of wires that penetrate the skin with a portion of them inserted in the body in close proximity to motor neurons [10, 11]. The electrodes are left in place temporarily while the stimulation is delivered; they are often used for short-term FES applications. Typical stimulation current amplitude used with percutaneous electrodes is also 25 mA.

##### Transcutaneous electrodes

Transcutaneous electrodes are placed on the surface of the body. They can be self-adhesive or can be secured to the skin with adhesive tape. During stimulation, the electrodes are placed over the nerve innervating the muscle to be stimulated. In addition to the fact that their use does not require surgery, transcutaneous electrodes can be repositioned immediately to ensure that the stimulation elicits the desired response (i.e., movement). Changing the position of the electrodes (and hence their effect) may be of particular importance if the stimulation needs to be modified in response to the changing needs of an individual, as is often the case during neurorehabilitation. This makes them ideal for temporary use, such as when using electrical stimulation as part of a short-term rehabilitation intervention. Also, the non-invasive nature of these electrodes makes it possible to use FES very early in the rehabilitation of patients who have had a stroke or an SCI, in which early intervention often leads to greater recovery [8]. The current used with transcutaneous electrodes (2–120 mA) is often greater than that necessary to produce muscle contractions with implanted ones. One of the important limitations of surface electrodes is that they may be unsuitable for use with deep muscles (i.e., far below from the skin); stimulation of muscles that are deep may require greater intensity which in turn may result in simultaneous contraction of undesired muscles.

#### Sensors for allowing user control

In addition to the characteristics of the electrical stimulator (e.g., number of channels, and programmable or non-programmable), the user interface for the neuroprosthesis may offer an additional level of customization. Neuroprostheses may accept commercially available accessible switches and, in some cases, it may also be possible to incorporate other specialized sensors allowing individuals with different abilities to command the device. For example, a goniometer can be used to trigger stimulation upon detection of wrist movements [12] and a potentiometer may allow the user to specify the grasp type to be produced through stimulation [13]. Specific examples are presented below.

#### Orthosis

The use of an orthosis can aid in the production of a specific movement when the stimulation alone is insufficient, by helping patients conserve energy or to prevent muscle fatigue. For example, an orthosis may be used to facilitate hand function [13] and provide stability while using electrical stimulation to facilitate walking [14–17].

## Technical considerations of FES: general stimulation characteristics

In addition to the stimulator, electrodes, user interface/control and control strategies, the characteristics of the actual electrical pulses used ultimately play a central role in the effects of the stimulation.

### Stimulation intensity: pulse amplitude and duration

**Fig. 1:**
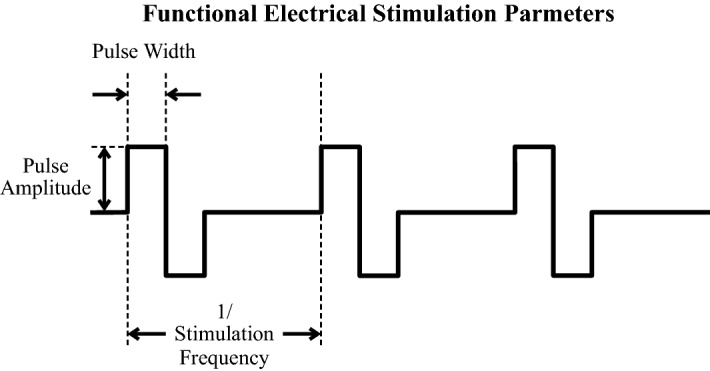
Functional electrical stimulation parameters. Pulse width, amplitude, and frequency define the muscles stimulated, force, and quality of the contraction

The intensity of the stimulation is determined by three parameters: pulse amplitude, pulse duration and pulse frequency (Fig. 1). The pulse amplitude refers to the magnitude of the stimulation. It affects directly the type of nerve fibers that respond to the stimulation with large fibers in close proximity to the stimulation electrode being recruited first [6]. The pulse duration (pulse width) is the time in which the stimulation pulse is present. Both parameters are inversely related so that an increase in pulse duration may require a pulse with lower amplitude to generate a response. Conversely, reducing the pulse duration may translate into the need to increase the amplitude of the stimulating pulse.

### Stimulation frequency

The frequency of stimulation is the rate at which stimulation pulses are delivered and it affects the strength of the muscle contraction as well as its quality. Each stimulation pulse with properly selected amplitude and duration produces a muscle twitch, characterized by a sharp increase in force followed by a slower return [18] to a relaxed state. Quick application of subsequent stimulation pulses before the muscle is relaxed will produce additional muscle twitches. The force produced by each twitch is added so that the mean force of the contraction is greater than that produced by a single twitch. Further increase in the pulse frequency results in a sustained contraction, in which no individual twitches are visible, and instead replaced by a smooth movement. This *tetanic* contraction is desired in FES applications. The minimum frequency required to induce fairly consistent contractions is between 16 and 20 Hz. However, while tetanic contractions can be achieved with a minimum of 20 pulses per second [8], a pulse frequency of 40 Hz is often needed. Higher pulse frequencies generate stronger tetanic contractions; however, they can also result in faster muscle fatigue. Pulse frequencies in the range from 20 to 50 Hz [6] are typical. For patients with SCI who often have problems with FES-induced muscle fatigue, in particular during early stages of FES use, pulse frequencies of 20–25 Hz are more common. In patients with stroke, for whom FES-induced muscle fatigue is not a major issue, we use a pulse frequency of 40 Hz.

### Pulse shape

**Fig. 2:**
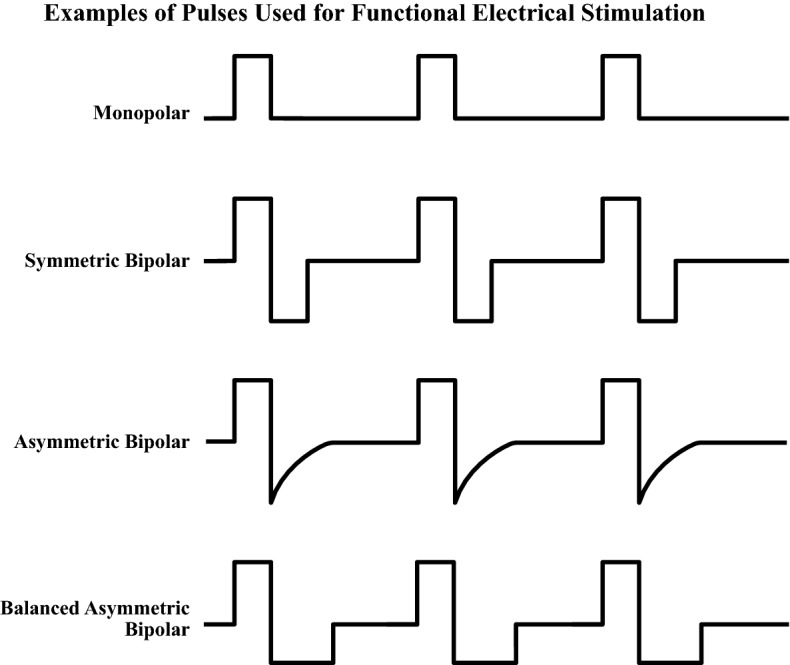
Examples of commonly used pulse shapes used for functional electrical stimulation (Modified from [6])

The pulses used for stimulation can be divided into monophasic and biphasic. In turn, biphasic pulses can be classified further into symmetric and asymmetric. It is believed that monophasic pulses can have a negative effect as they apply energy to the body that is never removed creating the potential to, among other things, damage the stimulated tissue. Biphasic pulses, on the other hand, alternate the anode and cathode electrodes with each stimulation pulse, which is safer for the person receiving the stimulation. *Symmetric biphasic* pulses, as the name suggests, consist of two phases which are identical in duration and amplitude with their polarity as the only difference between them. In contrast, *asymmetric biphasic* pulses also have two phases of opposite polarity but that are not identical in either amplitude and/or duration. However, in the case of *balanced asymmetric biphasic* pulses the amplitudes and durations of leading and trailing pulses are selected such that the total electrical charge delivered to the body during the leading pulse is identical to the total electrical charge removed from the body using the trailing pulse. This ensures the long-term safety and integrity of the stimulated tissues, while making it possible to control exactly which electrode is used to deliver stimulation that generates contractions and the exact motor point; by selecting the amplitude of the leading pulse to be sufficiently high to generate a desired muscle contraction and by selecting the amplitude of the trailing pulse to be sufficiently low not to trigger muscle contraction, one can deliver stimulation only to desired motor points with precision. Figure 2 displays examples of the different pulses commonly used for electrical stimulation.

## Practical aspects of FES

Before using FES, it is necessary to determine the values of the different stimulation parameters. Location of the electrodes and intensity of the stimulation are two of the most important aspects to consider when preparing to deliver stimulation. In combination, these parameters will determine the movement produced when the stimulation is delivered while ensuring the safety and comfort of the person receiving the stimulation.

### Electrode placement

The location of the stimulating electrodes has a direct impact on the muscles that are stimulated and, consequently, on the movements that are produced. Knowledge of functional anatomy is required to conduct this task as well as an understanding that each individual is unique. Identifying the optimal location of the electrodes often involves informed trial and error to verify that the facilitated movement is the desired one: electrodes are placed first over the nerve(s) innervating the muscle to be stimulated and stimulation is delivered. If the resulting movement is the desired one, the process is finished. Otherwise, electrodes are repositioned (usually using small adjustments of no more than a few centimeters) and the process is repeated until the desired movement is obtained.

### Intensity of the stimulation setting

The intensity of the stimulation will determine which muscles are contracted as well as the strength of their contraction. The process for determining the intensity of the stimulation is performed once the placement of the electrodes has been determined. As stated earlier, the intensity is determined by the pulse duration as well as the pulse amplitude. It is not uncommon to fix one of these parameters while adjusting the other one. For example, in the stimulators that we use both for research and therapeutic applications, namely Compex Motion [19] and MyndMove [20], a fixed pulse duration is commonly used while the pulse amplitude is adjusted to regulate the stimulation intensity. In this stage, the intensity of the stimulation is increased gradually often in increments as small as the stimulator allows. Several clinically important values can be identified during this exploratory ramping up of stimulation increases. These include

1. *Sensory threshold* is the lowest intensity in which the stimulation can be perceived by the person receiving the stimulation, even if no movement is produced.
2. *Motor threshold* is the minimum intensity that results in a visible muscle contraction, even if the contraction does not result in movement.
3. *Maximum tolerable intensity* is the maximum level that the person can tolerate without feeling discomfort.
4. *Operational stimulation intensity* is the intensity used to deliver stimulation during the actual use of FES.

### Clinical considerations for the use of electrical stimulation

Delivery of electrical pulses may affect tissue beyond the intended muscle with unexpected consequences. For this reason, it is important to mention a few important considerations and precautions prior to using electrical stimulation:

- Poor skin condition: Pressure injuries (a.k.a. pressure sores) or irritation prevents the use of self-adhesive electrodes.
- Poorly controlled epilepsy: When epilepsy is managed with medication with no seizure experienced for a reasonable period, FES can be used.
- A history of significant autonomic dysreflexia: Autonomic dysreflexia can be present in individuals with SCI above the sixth thoracic level (T6).
- Pregnancy: The effect of FES on the unborn child is not known in pregnancy.
- Cardiac pacemakers: Electrical stimulation may interact with the electrical signals from pacemakers interfering with their functioning.
- Cancerous tumor: Patients with a cancerous tumor in the area of the electrical stimulation should be excluded as potential tumor growth is a concern with the increased local blood flow resulting from the stimulation.
- Exposed metal: Patients with exposed orthopedic metal work should not receive electrical stimulation in the involved area.
- Unhealed fracture: Muscle contractions produced by FES around an unhealed fracture may result in a displaced fracture.
- Suspected, diagnosed, or uncontrolled Cardiovascular conditions: The cardiovascular demand resulting from the muscle contractions produced by the FES may require special attention prior and during delivery of stimulation.
- Botulinum toxin: Patients on botulinum toxin (e.g., Botox^®^) therapy for their upper limb, a procedure for reducing spasms after SCI, or that have received it in the last 6 months prior to the use of FES, may not respond to stimulation.

## Clinical applications of FES

FES has been used clinically as both an assistive device and a therapeutic intervention to facilitate restoration of volitional movement. This section describes systems that have played an important historical role in the development of FES as a rehabilitation technology. Examples of neuroprostheses for assistive applications are presented first, and include systems to facilitate standing, walking, grasping, and reaching and grasping. This list is followed by a presentation of seminal work that has helped establish the use of FES as a therapy. As in the previous case, the work described includes restoration of upper and lower limb function, and it is also categorized according to the target population (i.e., stroke and SCI).

### Functional electrical stimulation as an assistive device

One of the original motivations for the development of FES technology was to compensate for lost function. This application envisioned users wearing the device daily. By assisting in functions such as walking, standing, and grasping, the FES system would increase the user’s independence. Some historically important neuroprostheses are described next. Tables 2, 3 and 4 display a summary of the systems described in this section.

**Table 2:** Lower limb function neuroprostheses: standing and drop foot assistance

Function	Name	Target population	Number of channels	Orthosis use	Surface/implanted/percutaneous	FDA/CE approval	Control interface
Standing	Case Western Reserve University/Department of Veteran Affairs (CWRU-VA) Neuroprosthesis for Standing [17]	SCI	16	AFO	Implanted		
Walking with drop foot assistance	Odstock [22]	Stroke	1		Surface	FDA	Foot switch
	STIMuSTEP [23]	Stroke	2		Implanted	CE	Foot switch
	ActiGait [24]	Stroke	4		Implanted	CE	Foot switch
	NESS L300 [25]	Stroke	1		Surface	FDA/CE	Foot switch (force-sensitive resistor placed inside the shoe)
	Walk-aided foot drop stimulator [26]	Stroke	1		Surface	FDA	Tilt sensor or heel sensor

*SCI* spinal cord injury; *AFO* ankle–foot orthosis, *FDA* Federal Drug Administration, *CE* Conformité Européene

**Table 3:** Lower limb function neuroprostheses: walking with greater lower limb impairment

Function	Name	Target population	Number of channels	Orthosis use	Surface/implanted/percutaneous	FDA/CE approval	Control interface
Walking	Hybrid FES system developed by Andrews et al. [14]	SCI		AFO, spinal brace	Surface		Sensors on the handles of crutches, a spinal brace, or on the ankle–foot portion of the AFO Goniometers and FRS’s used by a state machine to determine the gait phase and time the stimulation
	Hybrid Assistive System [15]	SCI	6	Unilateral actuated orthosis	Surface		Switches, force transduces under the toe and heel, and potentiometers to measure knee and vertical shank displacement. All sensors used to define stimulation phase.
	Reciprocating gait orthosis (RGO) [28]	SCI	4	HKAFO	Surface		Push buttons placed on the handles of a walker
	Case Western Reserve University/Department of Veteran Affairs (CWRU-VA) Neuroprosthesis for Standing [17]	SCI	16	AFO	Implanted		Push buttons
	Parastep [29]	SCI	6		Surface	FDA	Push buttons placed on the handles of a walker
	COMPEX Motion FES system for walking [30]	SCI	4		Surface		Push button

*SCI* spinal cord injury, *AFO* ankle–foot orthosis, *HKAFO* hip–knee–ankle–foot orthosis, *FDA* Federal Drug Administration, *FSR* force-sensitive resistor

**Table 4:** Upper limb function neuroprostheses

Function	Name	Target population	Number of channels	Orthosis use	Surface/implanted/percutaneous	FDA/CE approval	Control interface
Grasping	Systems by Rebersek and Vodovnik [31]	SCI	3		Surface		Accommodates multiple sensors: (e.g., EMG, linear potentiometer, pressure sensors)
	Neuromuscular Electrical Stimulation System (NESS) H200 [13]	Stroke, SCI	3	Hinged wrist splint	Surface	FDA	Push button
	Bionic Glove [12]	SCI with active wrist function	3	Fingerless neoprene glove	Surface		Wrist position sensor
	Freehand System [32]	SCI	8		Implanted		Motion sensor mounted on opposite wrist or shoulder
Reaching and grasping	Belgrade reaching-grasping system [33]	SCI	4		Surface		Push button, accelerometer
	The COMPEX Motion Neuroprosthesis for Reaching and Grasping [19]	SCI	4		Surface		Accommodates multiple sensors
	MyndMove [20]	stroke, SCI	8		Surface	FDA	

*SCI* spinal cord injury, *EMG* electromyography, *FDA* Federal Drug Administration

#### Neuroprosthesis for standing

Among the functions that are often affected by SCI is the ability to stand. FES can be used to activate the muscles around the ankle joints which, in combination with a standing frame or a full-body orthosis to provide support to the trunk, can restore the ability to stand. Additional stimulation channels can also be used to facilitate trunk control. The Case Western Reserve University/Department of Veteran Affairs (CWRU-VA) neuroprosthesis for standing used a 16-channel implanted stimulator. Bilateral activation of the thighs, hip, and trunk allowed a person with paraplegia to stand upright for 8 min, when combined with an ankle–foot orthosis [17]. Electrodes were implanted bilaterally on the following muscles: gluteus maximus, posterior portion of adductor magnus, semimembranosus, tibialis anterior, tensor fasciae latae, quadriceps and sartorius. Intramuscular electrodes were used to activate trunk extensor muscles.

#### Neuroprosthesis for walking

The neuroprostheses used to facilitate walking after stroke and SCI are generally divided in two categories. The first one consists of devices that compensate for drop foot (described below), affecting ankle function. The second category is represented by neuroprostheses that can facilitate walking with greater impairments of the lower limb (e.g., bilateral impaired knee and hip flexion, in addition to limited ankle function) [8], often associated with SCI.

##### Neuroprosthesis for walking with drop foot

A common problem in individuals who have hemiplegia as a result of stroke is drop foot, a condition of decreased ability to perform ankle flexion and extension in the affected lower limb. As a consequence, the foot cannot clear the ground making it difficult or impossible to walk. Correction of drop foot using electrical stimulation is the simplest and oldest neuroprosthesis for walking. The procedure, first described in 1961 by Liberson et al. [21], is based on a single channel that stimulates muscles responsible for performing ankle dorsiflexion as well as the peroneal nerve eliciting a flexor reflex, which effectively eliminates the drop foot problem. Some examples of other neuroprostheses for walking with drop foot are.

Odstock [22]: delivers stimulation to the tibialis anterior muscle and peroneal nerve using a single stimulation channel and self-adhesive transcutaneous electrodes. A foot switch inside the user’s shoe triggers the stimulation and produces ankle dorsiflexion and eversion as well as a flexor withdrawal reflex (ankle dorsiflexion, flexion of hip and knee, and external rotation of the hip) in the swing phase of the gait. The FDA-approved device is commercialized by Odstock Medical Limited (Salisbury, Wiltshire, UK) and has been used by hundreds of individuals.

STIMuSTEP [23]: The STIMuSTEP is an implantable two-channel stimulator. One channel delivers stimulation to the deep branch of the peroneal nerve which produces ankle dorsiflexion. The second channel stimulates the superficial branch of the same nerve which results in foot eversion. Wireless communication is used to activate the stimulation which is timed using an external foot switch. This CE-marked (Conformité Européene) neuroprostheses was developed in the University of Twente and at Roessingh Research and Development (Enschede, The Netherlands).

ActiGait System [24]: Another implantable neuroprosthesis for walking with drop foot, this device uses a 4-channel stimulator and a 12-contact cuff electrode implanted close to the bifurcation of the peroneal nerve into the deep and superficial branches. Each electrode set can produce different ankle movements through stimulation of spatially (and functionally) distinct nerve fascicles. A foot switch synchronizes the stimulation with the gait using wireless communication between a transmitter and a receiver implanted in the user’s thigh. The ActiGait is commercialized by Ottobock SE & CO. KGAA.

NESS L300 [25]: Now commercialized under the name Bioness L300 Foot Drop System, this neuroprosthesis also stimulate the peroneal nerve. Synchronization of the stimulation and the gait is achieved through radiocommunication between the single-channel stimulator and a force sensitive resistor placed inside the shoe, underneath the foot.

WalkAide Foot Drop Stimulator [26]: Neuroprosthesis that also produces ankle dorsiflexion using a single-channel stimulation of the peroneal nerve and tibialis anterior muscle. Synchronization with the gait cycle is achieved using a tilt sensor or a heel sensor. The device, FDA-approved and invented in the University of Alberta (Edmonton, Alberta, Canada), is commercialized by Hanger Inc. (Austin, TX, U.S.A.).

##### Neuroprosthesis for walking with greater lower limb impairment

During the late 70 s and early 80 s, Kralj et al. [27] developed an FES method and system to facilitate walking in individuals with SCI. The system was designed to use a small number of stimulation channels to increase its clinical suitability and for easier use at home. The system used four electrodes placed bilaterally; for each leg one electrode was placed over the peroneal nerve and the second over the quadriceps muscle. Stimulation of the quadriceps locked the knees. Sudden stimulation of the peroneal nerve, after interrupting quadriceps stimulation, elicited the flexion withdrawal reflex which produced simultaneous ankle dorsiflexion as well as knee and hip flexion. This synergistic flexion, accompanied by upper body movements, facilitates the swing phase of gait. After this, the peroneal stimulation was interrupted and replaced with stimulation of the quadriceps completing a stride cycle. This sequence was repeated on the opposite leg resulting in gait. Users of this system were able to trigger the stimulation of each leg with two buttons placed on the handles of a walker or a pair of crutches used for balance and safety. This technique continues to be used today with variations that include additional electrodes. Other examples of neuroprostheses for walking with bilateral severe lower limb impairment include:

Hybrid FES system developed by Andrews et al. [14]: The system developed by Andrews and his colleagues also combines peroneal and quadriceps stimulation with instrumented passive braces. As with other similar devices, the user could trigger walking with sensors placed on the handlebar of a rolling walker, a spinal brace, and a ground (floor) reaction ankle–foot orthosis (AFO). In addition, goniometers and force-sensitive resistors were used by a finite state rule-based controller that could estimate the gait phase and trigger the stimulation on each leg accordingly.

Hybrid Assistive System (HAS) [15]: developed by Popovic et al., the HAS combined FES with a modular actuated orthosis (self-fitting modular orthosis—SFMO). A shared rule-based controller synchronized the activation of the FES system and the SFMO. This resulted in the FES being used only to produce movement while stabilization was achieved by the orthoses.

Reciprocating Gait Orthosis (RGO) [28]: Also known as the Louisiana State University Reciprocating Gait Orthosis (LSU-RGO), the device was developed in the 1970s at the Louisiana State University for children with lower extremity musculoskeletal disabilities (e.g., spina bifida, muscular dystrophy, etc.) and was later tested with adults with SCI during the late 1970s and early 1980s. The device was an electromechanical hybrid system that combines a passive reciprocating hip–knee–ankle–foot orthosis (HKAFO) and four-channel electrical stimulation. Reciprocal movements of the legs were enabled by a (mechanical) cable that connected the hip joints of the orthosis; flexion of one hip resulted in extension of the contralateral hip. Stimulation was used to produce hip flexion and contralateral hip extension through simultaneous stimulation of the rectus femoris muscle of one leg and contralateral hamstrings. Use of the RGO also required a rolling walker on which switches were mounted for users to trigger the stimulation.

CWRU-VA [17]: In addition to standing, the CWRU-VA implanted system (mentioned above) was also used to restore walking. The implanted sensors increased the selectivity of the stimulation making it possible to stimulate hip flexor muscles in isolation, which can produce a more natural gait.

Parastep [29]: The Parastep is a neuroprosthesis designed to facilitate walking in individuals with paraplegia resulting from SCI that uses Kralj’s technique based on peroneal stimulation. The system, developed in the mid to late 1990s, uses six pairs of self-adhesive electrodes connected to a microcontroller-based electrical stimulator attached to the users’ belt. Electrodes are placed over the right and left peroneal nerves, quadriceps, and if necessary, paraspinals or gluteus maximus/minimus. Buttons on the handles of a walker let the user activate the stimulation. The system allows its users to ambulate between 6 and 9 m with a few users reported walking over 800 m. Now in its Parastep I version, the system is FDA-approved and is commercialized by Sigmedics Incorporated (Chicago, IL, USA).

COMPEX Motion FES system for walking [30]: Developed by Thrasher et al., the system did not elicit the withdrawal reflex through peroneal stimulation. Instead, it used 8 electrode pairs to deliver bilateral stimulation to the quadriceps, hamstrings, gastrocnemius/soleus, and tibialis anterior. The user pressed a button to initiate a stimulation sequence that would produce a step: first, the hamstrings were stimulated. Almost simultaneously, stimulation of the tibialis anterior was activated although with a lower rate of amplitude increase (it took longer to reach its maximum amplitude). After this, stimulation to the hamstrings was ended followed by the tibialis anterior. At the same time, quadriceps stimulation was slowly ramped up and finally followed by activation of the gastrocnemius. Quadriceps and gastrocnemius stimulation was used during the mid and late stand phases of gait. This system was tested with the help of five individuals with SCI, four with thoracic injuries and one with a cervical level of injury. Of these participants, three could walk with a rolling walker and two using two canes, with one also requiring a knee–ankle–foot orthosis (KAFO).

#### Neuroprosthesis for upper limb function

FES systems have also been created to facilitate upper limb function including reaching and grasping. As with case of FES systems to assist standing and walking, neuroprostheses for reaching and grasping can be implanted or non-invasive. They are often designed to produce grasping as well as releasing, thus making manipulation of objects possible.

##### Neuroprosthesis for grasping

Examples of neuroprostheses for grasping include

Systems by Rebersek and Vodovnik [31]: Developed in the early 1970s at the University of Ljubliana (Ljubliana, Slovenia), the three-channel stimulator could be controlled with different sensors including ones for electromyographic (EMG) recording and a sliding resistor which controlled the stimulation intensity. A design that offers the capacity to select an optimal interface to match the users’ physical abilities is one of the most important contributions of this system.

Neuromuscular Electrical Stimulation System (NESS) H200 [13]: Originally called the NESS Handmaster, the device has three stimulation channels that, like other neuroprostheses for grasping, stimulate finger extensors and flexors, as well as the thumb. The stimulation electrodes are placed on the inside of a carbon fiber splint which provides support to the hand and wrist. The hinged splint simplifies the donning and doffing of the device. Stimulation intensity and duration can be adjusted by the user with an external control box which also allows selection of the stimulation pattern. A potentiometer in the same control interface makes it possible to define the control position to facilitate different grasping styles. Triggering of the stimulation is achieved using either a button on the control box or one placed directly on the splint. The device has been commercially available for decades and has been used by stroke survivors with hemiplegia as well as individuals with tetraplegia resulting from SCI. An FDA-approved updated version of the system is available under the name H200 Wireless Hand Rehabilitation System (Bioness, Inc., California, USA).

Bionic Glove [12]: Originally developed in 1989 at the University of Alberta, Edmonton, Canada, the bionic glove consists of a fingerless glove equipped with a wrist position sensor as well as an electrical stimulator. It is designed for individuals with SCI with active wrist flexion and extension. The glove is worn over surface electrodes that stimulate muscles to extend and flex the fingers and thumb. Extending the wrist triggers stimulation to produce a grasp (similar to tenodesis grasp) while wrist flexion results in opening of the hand.

The Freehand System [32]: Also developed at the Case Western Reserve University, the Freehand was an implanted neuroprosthesis with eight epimysial electrodes (i.e., attached surgically to the muscle surface) that controlled extension and flexion of the finger and thumb to assist individuals with SCI. One of the electrodes was implanted in an area with intact sensory function and used to provide sensory feedback to the user. The device’s power and control signals were provided from an external source (i.e., not implanted). The user could activate the device with movements of the wrist or opposite shoulder, which were detected with a motion sensor mounted externally. Different positions of the shoulder could be used to trigger hand opening or closing and fast shoulder movements allowed the user to sustain the stimulation until the next fast movement. The Freehand system was FDA-approved and commercialized by the company NeuroControl Corporation.

##### Neuroprosthesis for reaching and grasping

In addition to devices that can produce grasping, there are also a few neuroprostheses that have the capability of facilitating reaching as well. This is important as both movements are often used synergistically, and for individuals with high levels of impairment, it may be necessary to produce both actions using electrical stimulation. Specific devices include:

The Belgrade Grasping-Reaching System [33]: This noninvasive neuroprosthesis generated palmar and lateral grasps, which the user could select through the use of a button, as well as hand opening using three stimulation channels. The fourth channel was used to stimulate the triceps brachii muscle and it was triggered by the angular velocity of the shoulder.

The COMPEX Motion Neuroprosthesis for Reaching and Grasping: This four-channel programmable neuroprosthesis was designed specifically to facilitate reaching and grasping in individuals with SCI [19]. It was a direct evolution of the neuroprostheses developed at by the Automatic Control Laboratory at the Swiss Federal Institute of Technology Zurich (ETHZ) and the Paraplegic Center at the University Hospital Balgrist (ParaCare) [8]. Every aspect of the stimulation (e.g., amplitude, duration, frequency, ramp up, ramp down, etc.) could be programmed using a desktop software and loaded into the device using chip cards. This made it possible to implement any reaching and grasping movement that could be produced with surface electrical stimulation. This device has even been used for producing gait [30], as mentioned above. In addition, the system could be triggered by numerous sensors; the stimulator was equipped with digital and analog inputs that could be used to create customized interfaces to match the functional abilities and requirements of every user.

MyndMove: One of the newest commercially available stimulators is the MyndMove, which was created specifically to use FES as a therapeutic intervention to restore upper limb function after stroke and SCI. MyndMove systems has evolved from the work and results achieved with the Compex Motion systems. The system offers more than 30 reaching and grasping protocols, including some that can facilitate bimanual tasks as well as fine finger manipulations. The device has been used successfully to restore upper limb function in individuals with chronic severe hemiplegia [20], SCI, traumatic brain injury, cerebral palsy, cervical myelopathy and brachial plexus injuries.

### Functional electrical stimulation as a therapeutic intervention for restoring voluntary movement

#### Carry-over effect: The use of FES as a treatment

Anecdotal reports of a therapeutic effect existed ever since the first FES devices to assist drop-foot in individuals with hemiplegia were introduced by Liberson [21] in the early 1960s [34]. More specifically, users of FES systems reported that their ability to control their muscles voluntarily had improved even without using the neuroprosthesis. Generalized use in the 1970s of neuroprosthetic devices to facilitate activities of daily living led to the further realization of a *carry*-*over effect*. This gave rise to the use of FES technology as a short-term intervention in which users underwent therapy using the neuroprosthesis which resulted in patients with stroke regaining the ability to move voluntarily [9, 35].

Alongside the development of new FES technology that made it possible to create new neuroprostheses with increased clinical viability, research in the 90 s started the systematic investigation of the carry-over effect, leading to the eventual use of FES as a therapeutic intervention. Where traditionally FES was used as an assistive device for the remainder of an individual’s life, FES can now be used as part of a short-term intervention with the goal of restoring a person’s ability to move independently, without assistance from the neuroprosthesis. This is known as functional electrical stimulation therapy (FEST). Today, FEST has become one of the most important tools available to therapists dedicated to the neurorehabilitation of individuals who have sustained a stroke or SCI.

#### FEST intervention

**Fig. 3:**
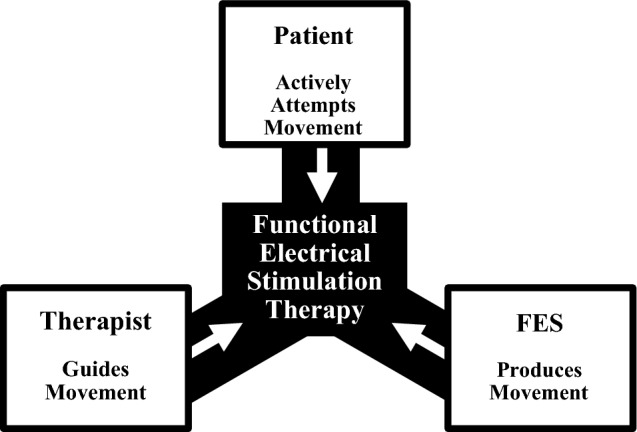
Fundamental components of functional electrical stimulation therapy. FEST has three components. First, a patient must be actively attempting a motor task. Second, an FES system produces the intended movement which also generates the corresponding correct sensory feedback. Third, a therapist guides the limb in motion to ensure the quality and correctness of the movement. The therapist also adjusts the stimulation according to the changes observed in the patient throughout rehabilitation

In an FEST intervention, patients are often asked to attempt functional movements with their affected limb(s), and after a few seconds of trying, a therapist triggers the stimulation specifically designed to produce the attempted movement. This procedure is repeated multiple times in each session in which multiple movements are often practiced. Although interventions can vary in duration, one of the most common number of sessions for FEST research is 40. As the ability to perform voluntary movements is restored, use of the FES is gradually reduced until it is discontinued completely at the end of the intervention. There are three fundamental components of FEST. First, the individuals receiving the therapy must attempt the movement (often achieved by asking them to complete a functional motor task). Second, the electrical stimulation facilitates the practiced task producing the motor response and the correct and congruent sensory feedback. Stimulation in this context is used only for the movements that the user is unable to produce volitionally. Third, a therapist guides the limb in motion to ensure the correctness and quality of the movement. The therapist also modifies the electrode placement and stimulation parameters as appropriate, based on the user’s progress during the FEST intervention [36] (Fig. 3). It is believed that the repeated simultaneous presence of a patient’s intention to move and the sensory feedback resulting from the FES-assisted movement produce neuroplastic changes that ultimately lead to the restoration of voluntary motor function [36].

##### Interventions to restore voluntary movement after stroke and spinal cord injury

FEST has been used successfully to restore upper and lower limb voluntary movement in individuals who have sustained a stroke or SCI. The next sections describe some of the initial reports that have helped develop FES as a therapeutic intervention.

i. *Restoration of lower limb function using FEST after stroke*
  Some of the earliest reports of a carry-over effect were noted after a few applications of the use of a neuroprosthesis to correct drop foot, allowing individuals with stroke to improve their ability to perform dorsiflexion [9, 35]. In the same decade, in some of the first experiments to formally investigate the carry-over effect, Merletti and his colleagues compared the effects of FES to produce ankle dorsiflexion through stimulation of the peroneal nerve [37] against ‘traditional physiotherapy treatment’. The torque produced by voluntary dorsiflexion of the ankle was three times higher in the treatment group when compared to the control group. Taylor and his colleagues reported an increase in walking speed after using a drop foot stimulator for 18 weeks [22]. Important evidence was provided in 2010 by Stein et al. [26] who reported an increase in walking speed in 41 individuals in the chronic stages of stroke rehabilitation. Individuals used a drop foot stimulator in the community for up to 1 year. There is considerable evidence that using an FES system to assist walking with drop foot results in sustained improvements in lower limb function.
ii. *Restoration of lower limb function using FEST after SCI*
  In 1999, Badj et al. [38] provided the first documented evidence of FEST used for lower limb rehabilitation after SCI. In their work, they demonstrated an increase in strength and improvement in drop foot and plantar flexion after training using a neuroprosthesis for walking. Thrasher and colleagues measured the efficacy of FEST to restore the ability to walk in individuals with chronic incomplete SCI, a population for which functional changes are typically unexpected [30]. Five individuals with cervical and thoracic injuries completed a 12- to 18-week intervention in which FEST was applied to at least one of their legs during assisted walking. Details of the stimulation used can be found above (Compex Motion FES System for Walking section). At the end of the rehabilitation, four participants increased their stride length and stepping frequency resulting in an increase in speed. The changes in the 5th participant allowed her to decrease her reliance on an assistive device to walk. In a more recent study, Kapadia et al. [39] conducted a phase I randomized control trial in the same population (chronic, incomplete SCI between C2 and T12 levels). The neuroprosthesis and protocol were identical to the ones used by Thrasher et al. [30]. The study revealed that the locomotion function improved significantly more with FEST than a non-FEST controlled intervention.
iii. *Restoration of upper limb function using FEST after stroke*
  The positive effects of FEST on upper limb function for the rehabilitation of individuals who have had a stroke has been documented for decades. After witnessing evidence of the benefits of using FEST for increasing muscle strength in individuals with hemiplegia in 1996 [40], several studies focused on the effects of the intervention to restore reaching and grasping function in both acute [41–46] and chronic [46–52] stroke populations. It is important to mention that FEST has been demonstrated to be effective in the rehabilitation of individuals with chronic severe hemiplegia resulting from stroke [20, 44], a population for whom rehabilitation is traditionally considered to have little to no effect.
iv. *Restoration of upper limb function using FEST after SCI*
  Popovic et al. [53] demonstrated the efficacy of the Bionic Glove that, after 6 months of use, improved upper limb function (increased power grasp and/or range of movements) in individuals with tetraplegia resulting from SCI at the C5–C7 level. This represented the first concrete evidence of the restorative effects of FEST for upper limb function in individuals with SCI.
  In 2005, Mangold and her colleagues reported improvements in grasping function or muscle strength in the majority of 11 individuals who received FEST using a neuroprosthesis for grasping [54]. The participants of that study had sustained an SCI at the C4–C7 levels and were in the acute and subacute stages of rehabilitation.
  More recently, Popovic and his colleagues [55] compared FEST and conventional occupational therapy (OT) to restore upper limb function in individuals with cervical SCI (C3–C7). Each of the 21 participants received a one-hour session of OT followed by either one additional hour of OT (control group) to address grasping or one hour of FEST (treatment group). After 40 sessions, the intervention group experienced significantly larger improvements in function and the grasping effects endured, and in some cases improved, months after the intervention. The same research team reached a similar conclusion when applying this therapy to restore grasping function in individuals with incomplete SCI (C4–C7) in the chronic stages (more than 24 months after their injury) of rehabilitation [56].

## Future directions of FES

Some of the new directions in which FES can evolve will likely focus on its therapeutic effects, its technology, and strategies to deliver stimulation as well as its applications. Two important examples are described below.

### Brain–computer interface-driven FES

An important direction for FES research and applications is its integration with brain–computer interfaces (BCI), devices that can translate brain signals into control commands. The main rationale for integrating BCI and FES devices for neurorehabilitation is that the simultaneous presence of a motor command (resulting from a person’s attempt to move) and sensory information (proprioceptive and somatosensory) resulting from the FES-produced movement will promote changes in the nervous system that will ultimately lead to restoration of voluntary movement [57]. BCI systems provide the opportunity to identify the intention to move even when no overt movements are present due to the severity of the impairment. The combination of the two technologies has opened the door to the development of several new interventions which are currently under investigation by researchers around the world. One of the earliest reports on the integration of noninvasive FES and BCI technology described a system to restore hand function in a person with tetraplegia resulting from a traumatic SCI sustained 5 years before the date in which the report was published [58]. The FES system was designed to produce lateral grasp and hand opening of the left hand. The BCI was implemented as a brain switch that was triggered by the participant imagining foot movements, which produced a unique power increase in the 17 Hz (beta range) frequency band of his electroencephalograpic (EEG) activity. Each activation of the BCI produced a state transition in the following sequence: (1) finger extension (hand opening), (2) finger closing, (3) closing of the thumb, and (4) finger extension. A 5-s refractory period was used between each phase transition (BCI activation) to prevent undesired quick activations. After these steps, the system would enter an idling state and was ready for a new cycle to start. Later, the same group presented the control of an implanted FES system for grasping [59]. That report described the system used by another individual with tetraplegia (C5 complete) who had been implanted with a Freehand system in his right upper limb, which was used to facilitate lateral grasp. The stimulation sequence consisted of (1) finger extension (hand opening), (2) finger closing, and (3) closing of the thumb. The participant was able to control the FES system using imagination of his left hand. It is significant to note that in this case the participant was able to achieve control over the device after only 3 days of training, while other systems at the time required multiple sessions, decreasing their clinical appeal. In a proof-of concept study, Marquez-Chin [60] reported on the control of a neuroprosthesis for grasping using off-line classification of electrocorticographic (ECoG) signals to trigger different grasps (lateral and palmar). A person with SCI (C6) was fitted with the FES system and, using a button, would select the ECoG activity of a trial (selected at random with each button press). The ECoG signal had been previously recorded from a second person implanted with subdural electrodes, while performing different arm movements (reaching to the right, left, or flexing the wrist). The system would then classify the randomly selected signal and trigger a pre-assigned movement with the neuroprosthesis (i.e., correct classification of the ECoG signals would trigger the pre-determined hand movement). These early demonstrations were important as they showcased the integration of two important technologies for motor restoration and their focus was primarily on the use of this technological combination as an assistive device. The notion of using BCI and FES technologies together as a therapeutic intervention to restore voluntary movement after paralysis was developed later. In recent years, there has been an increase in reports describing the combination of FES and BCI technologies focused on the neurorehabilitation of people with stroke and SCI. Daly et al. [61] described the use of a BCI-triggered stimulation system to facilitate extension movement of the right index finger in a woman 10 weeks after sustaining a stroke that resulted in losing the ability to perform isolated finger movements. The BCI was designed to trigger index finger movements after identifying the participant’s imagined or attempted index finger movements. After nine sessions, the participant’s ability to extend her affected index finger at the metacarpophalangeal joint increased 26°. This study was one of the first ones to demonstrate joint BCI and FEST used to restore voluntary movement after stroke. In 2016, Marquez-Chin et al. [62] described the use of an EEG-triggered FES system to restore upper limb function in a person with chronic severe hemiplegia. The participant of that study had sustained a stroke 6 years before the study during which he had tried multiple interventions, including FEST alone, all of which had failed to produce any significant improvements in his arm and hand function. After 40 one-hour sessions of therapy, in which five different reaching movements were practiced (reaching forward, to the knee, to the mouth, and to the opposite shoulder as well as lateral reaching), the participant experienced clinically significant changes in his upper limb function. More recently, the same group tested the EEG-triggered FEST to restore the reaching and grasping function of a second individual, also with chronic severe hemiplegia resulting from a stroke 6 years before the study and who, like the previous case, had tried multiple interventions to improve his voluntary movement unsuccessfully [63]. The intervention consisted of 80 one-hour sessions delivered three times per week. In each session, multiple reaching and grasping techniques were practiced. At the end or the intervention, the participant’s upper limb function showed clinically significant and meaningful changes.

### Textile-based FES

**Fig. 4:**
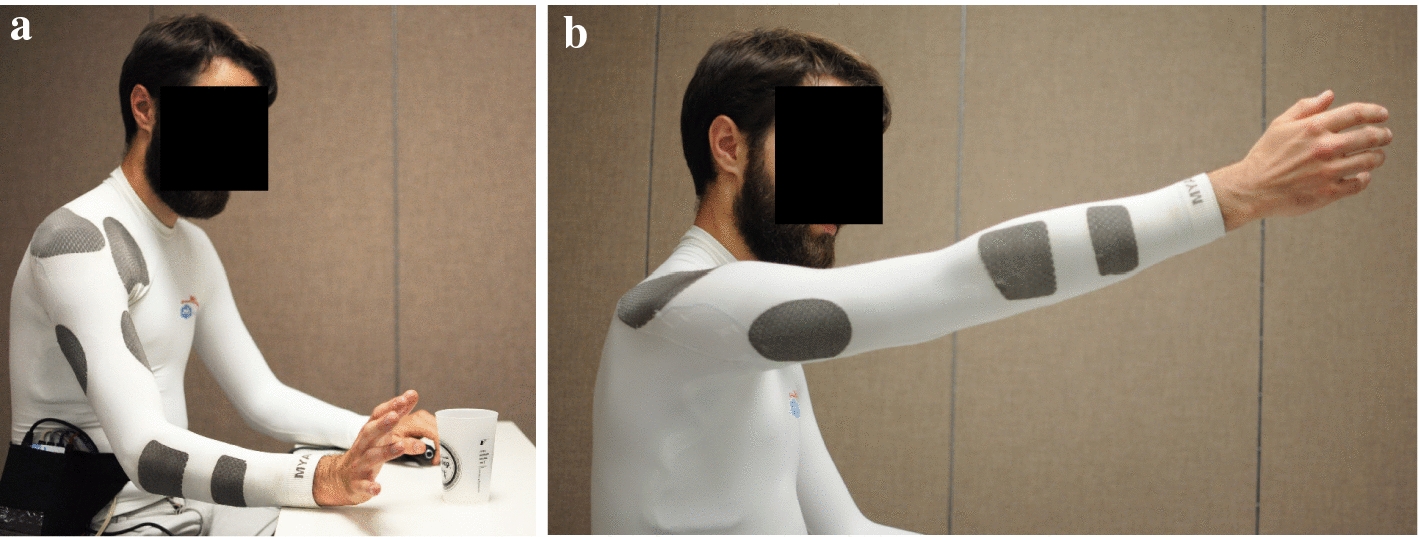
Textile-based neuroprostheses. **a** Finger extension produced using a shirt designed for implementing a neuroprosthesis for reaching and grasping. The garment includes rectangular areas (dark grey patches) made of conductive yarn that function as electrodes. **b** Forward reaching. Details can be found in [65]

Although more commonly used for recording biological signals, there has been a recent increased interest in textile-based delivery of FES. Innovations in textile technology have made it possible to create garments that combine cloth with sections incorporating conductive yarn. Potential advantages of textile-based electrodes include increased user comfort, better mechanical compliance, no skin irritation due to increased ventilation (when compared to hydrogel-based electrodes), and the possibility of washing them [64]. More importantly, textile-based garments can also be integrated into regular clothing which has the potential to simplify the delivery of FES and, consequently its use. This is further supported by the fact that garments can be mass-produced [65]. Examples of textile-based electrodes include the Smart Electrode and the Smartex [66]. Yang and colleagues developed an electrode array with the electrodes screen-printed onto a polyester/cotton fabric [67]. The design consisted of four layers, including a first one to serve as a substrate on the fabric for further printing (referred to as the interface layer), a silver conductive layer containing the conductive pads and tracts, an encapsulation layer providing electrical insulation and protection, and a carbon-loaded silicone layer over the conductive pads and in contact with the skin, which made the use of the electrodes without connective gel possible. The electrode was used to produce pointing with the index finger, precision pinch, and hand opening (finger extension) in two healthy participants. Recently, Moineau et al. [65] reported on the design and testing of shirt and leggings that were specifically designed to produce upper and lower limb movements. The garments were constructed with nylon and spandex with the addition of patches including a nylon yarn coated with silver/silver chloride and spandex. These patches functioned as electrodes (Fig. 4). The elasticity of the materials used resulted in garments that are able to generate compression maintaining the position in place as well as proper contact with the skin. The garments included eight electrodes as part of their construction which were able to deliver stimulation to finger flexors, biceps, triceps, as well as anterior and posterior deltoid. The garments are now undergoing clinical testing with individuals who have had a stroke or a spinal cord injury.

Despite the demonstrated feasibility of using textile-based electrodes to deliver FES, one important aspect that is being investigated is the comfort of the individual receiving the stimulation using this technology. Zhou et al. [64] reported in 2015 on the comfort of stimulation when using a rectangular electrode made with silvered thread to deliver stimulation to the tibialis anterior muscle resulting in foot dorsiflexion. In their study, they found a wet textile-based electrode was able to produce movement, similar to a hydrogel, while applying stimulation with a dry textile-base electrode produced pain even at low stimulation currents. Similar observations were reported by [65] when using their garments for FES delivery.

## Conclusions

FES has provided tangible assistance to individuals with mobility impairments for decades. The technology has allowed participation in daily life which otherwise would be difficult or impossible. Today, FEST is an important tool available to therapists working in the field of neurorehabilitation. The evolution of the technology has facilitated its use in clinical environments and has already produced some of the largest improvements in motor function of individuals with stroke and SCI. The prevalence of FEST will likely increase in the next decade with an expected increase in the aging population; age is a risk factor of stroke. It is possible that with this change in demographics, the emphasis of stroke care will change to continue to include prevention and cure, with a newly increased focus on rehabilitation as well [68]. Significant challenges lie ahead including increasing the efficacy of FEST as well as its adoption. Currently, the majority of FES systems still require that users delivering the intervention have knowledge of functional anatomy, as well as the effects of each of the stimulation parameters on the contraction produced by the stimulation. This is particularly true for an intervention in which the use of FES is modified according to the patients’ response to therapy; progressively challenging and/or complex movements are common in FES therapy as well as a gradual decrease in its use as recovery of voluntary motor function takes place. New technologies will play a fundamental role in a larger adoption of FES. Inclusion of new stimulation form factors, such as the wearable systems described here, along with automatic optimization of stimulator parameters will likely play a central role in the increased use of FEST. In addition, the combination of FEST, along with the further inclusion of new understanding of the neuroplastic basis of motor learning, provides an opportunity to see further increases in the efficacy of this intervention.

## Data Availability

Not applicable.

## Abbreviations

BCI: Brain–computer interface
EEG: Electroencephalography
ECoG: Electrocorticography
FES: Functional electrical stimulation
FEST: Functional electrical stimulation therapy
NMES: Neuromuscular electrical stimulation
SCI: Spinal cord injury
CE: Conformité Européene
